# Relevant Cytokines in the B Cell Lymphoma Micro-Environment

**DOI:** 10.3390/cancers12092525

**Published:** 2020-09-05

**Authors:** Günter Krause, Floyd Hassenrück, Michael Hallek

**Affiliations:** 1 Department I of Internal Medicine, University of Cologne, Kerpener Str. 62, 50937 Köln, Germany; floyd.hassenrueck@uk-koeln.de (F.H.); michael.hallek@uni-koeln.de (M.H.); 2 Cologne Cluster of Excellence on Cellular Stress Responses in Aging-associated Diseases (CECAD Cologne), Joseph-Stelzmann-Str. 26, 50931 Cologne, Germany

**Keywords:** chronic lymphocytic leukemia, T cells, lymphoma-associated macrophages, chemokines, B cell receptor, B cell receptor-associated kinases, PI3K, BTK, idelalisib, ibrutinib

## Abstract

Cytokines are soluble protein factors with importance in intercellular communication and, as such, play pivotal roles in the pathogenesis of B cell malignancies. Evidence from in vitro cultures permitted us to choose example cytokines that bind to different biochemical receptor types. Activated malignant B cells or stromal fibroblasts and macrophages prominently secrete the chemokines CCL3 or CXCL12 and CXCL13, respectively. Apart from helper T cells, various cell types of the B cell lymphoma microenvironment are capable of producing the cytokines IL-4, IL-6, IL-10 and TNFα. Owing to its impact on the development of myeloid cells, CSF-1 is among important soluble factors in the B cell lymphoma microenvironment. Inhibitors of B cell receptor-associated kinases often act via the blockade of cytokine production, but also prevent cytokine effects, e.g., chemotaxis. Increments in blood levels in chronic lymphocytic leukemia patients compared to healthy donors and normalization upon treatment with ibrutinib can be explained by producing cell types and modulation of cytokine production observed in vitro.

A recent review article about cytokines in chronic lymphocytic leukemia (CLL) [1] prompted us to provide an overview of the current understanding of the biological basis of cytokine production by various cell types and how this is reflected or not in the cytokine blood levels observed in CLL patients compared to healthy controls. For this purpose, we wish to outline the biological diversity of cytokines involved in B cell malignancies, the roles of selected cytokines in the micro-environmental dialogue, and how these roles may influence clinically observed blood levels of the respective cytokines.

## 1. Biological Diversity of Cytokines

Talking about intercellular communication by soluble protein factors in quite general terms, it is of advantage to stratify the involved cytokines according to relevant categories. While producing or affected cell types often are difficult to define and alterations of cytokine blood levels may be influenced by multiple factors, biochemical receptor types offer a systematic classification scheme. Apart from the ligands of the class named catalytic receptors (CatR) that include a family called cytokine receptors (CkR) as well as e.g., tumor necrosis factor receptors (TNFR) and receptor tyrosine kinases (RTK) [2], cytokines may also belong to other categories, for instance chemokines that bind to G-protein-coupled receptors (GPCR). The interleukin (IL) nomenclature system was a useful innovation four decades ago, but the concept of ILs is increasingly superseded by that of cytokines that also include cellular interactions of cell types beyond leukocytes. Many, but not all of the cytokines that are named IL bind to members of the CkR family, which commonly signal via the JAK/STAT pathway. However, this heterogeneous group also includes, for instance, IL-8 and IL-34 that, according to their receptors, belong to other cytokine subgroups, namely chemokines and growth factors, respectively. Using the full spectrum of candidate cytokines without restriction to so-called ILs, unlike a recent review article [1], we selected examples of cytokines with relevance in B cell malignancies based on in vitro experiments involving lymphoma and bystander cells. After examples of chemokines that are predominantly produced by activated tumor cells, stromal fibroblasts or macrophages, we discuss cytokines that can be secreted by T cells and other cell types [3,4] and talk about myeloid bystander cells and the impact of colony stimulating factor 1 (CSF-1) on their development. Possible associations of cytokine production in the lymphoma microenvironment with corresponding clinically observed blood levels in disease versus healthy controls and upon treatment with inhibitors of B cell receptor (BCR)-associated kinases (BAK) are examined in a systematic and quantitative manner.

## 2. Chemokine Production by Activated Malignant B Cells

The chemokine CC-motif ligands (CCL) 3 and 4 are produced by CLL cells in co-culture with so-called nurse-like cells and upon engagement of the B cell receptor (BCR) [5]. Inhibitors of BAK, namely idelalisib and ibrutinib, which target PI3Kδ and BTK, respectively, block this chemokine secretion by CLL cells [6,7]. Ibrutinib at concentrations above 10 nM inhibits the secretion of CCL3 and CCL4 by anti-IgM-stimulated Ramos. The C481S mutation in BTK rescues the chemokine secretion in the presence of up to 1 µM ibrutinib [8].

CCL3 and CCL4 serve as chemo-attractants of T lymphocytes and monocytes and lead to infiltration of these cell types into bone marrow and lymph nodes [9,10]. Therefore, maintained cytokine production in the presence of ibrutinib owing to the C481S mutation suggests that CCL3 and CCL4 may shape a supportive lymphoma microenvironment and thus contribute to in vivo selection of malignant B cells that express mutant BTK [8].

In accordance with its production by activated lymphoma cells, CCL3 is one of the most prominent cytokines with increased blood levels in CLL patients compared to healthy controls and dominates the cluster analysis of a comprehensive cytokine panel [11]. In accordance with ibrutinib-sensitive cytokine production by activated lymphoma cells, the elevated blood levels of CCL3 and CCL4 in CLL patients show strong reduction upon ibrutinib treatment compared to other cytokines (Table 1) [12].

## 3. The Homing Chemokines CXCL12 and CXCL13

The chemokine CXC-motif ligand 12 (CXCL12), formerly named stromal cell-derived factor 1 (SDF1), represents one of the most prominent and first recognized examples of cytokine-mediated interactions of CLL and bystander cells in co-cultures [13,14]. Stromal fibroblasts and adherent cells that grow out from peripheral blood mononuclear cells constitutively secrete CXCL12, which supports the survival and localization of CLL cells in lymphoid tissues. Since differentiated THP-1 macrophages, which provide similar survival support to CLL cells as bone marrow-derived murine macrophages [15], do not secrete CXCL12 [16], it is not clear whether production of this chemokine can be unequivocally attributed to myeloid bystander cells. In contrast, production of CXCL13, also known as B cell-attracting chemokine 1 (BCA-1), is linked to CD68^+^ macrophages by immunocytochemistry [17]. Secretion of the predominantly macrophage-derived CXCL13 [18] in bone marrow aspirates from CLL patients is significantly decreased during treatment with ibrutinib [12].

Idelalisib and ibrutinib inhibit migration of CLL cells to the homing chemokines CXCL12 and CXCL13 [6,7]. This provides an explanation for the compartment shift from lymphoid tissues into blood that often occurs early during treatment with these drugs. Compared to healthy donors, the blood levels of CXCL12 or CXCL13 in CLL patients are decreased or increased, respectively [17,19], which may reflect predominant production of these homing chemokines by stromal fibroblasts or myeloid bystander cells (Table 1). Elevated CXCL13 levels in CLL patients normalize during ibrutinib treatment [20].

**Table 1:** Selected cytokines of the B cell lymphoma micro-environment and alterations of their plasma levels.

Cytokine	Receptor Type ^1^	Producing Cell Type ^2^		Blood Levels in CLL Patients ^3^ (-Fold of Reference)	
				CLL vs. Healthy Controls	Ibrutinib vs. Untreated CLL
CCL3	CCR1, *CCR* **GPCR**	act. B-L [5]		6.7, **** [11]	0.1, **** [12]
CCL4	CCR5, *CCR* **GPCR**			1.7, **** [11]	0.3, **** [12]
CXCL12	CXCR4, *CXCR* **GPCR**	stromal fibroblasts [13]		0.5, ** [19]	n.d.
CXCL13	CXCR5, *CXCR* **GPCR**	mon. ph. [18]		3.9, *** [17]	0.3, *** [20]
IL-4	Type I *CkR* **CatR**	T lymphocytes [21]	CLL-enriched CD8^+^ subset [22]	1.6, n.s. [11]	0.6, n.s. [12]
IL-6	Type I *CkR* **CatR**			2.8, * [11]	0.5, n.s. [12]
IL-10	Type II *CkR* **CatR**		act. B-L [23] M2 Mθ [24]	0.8, **** [11]	0.4, **** [12]
TNFα	*TNFR* **CatR**		mon. ph. [25] M1 Mθ [24] act. B-L [8]	4.2, * [11]	0.3, * [12]
CSF-1	CSF1R, *RTK* **CatR**	adherent mon. ph. [25] act. B-L [26]		2.7, **** [27]	n.d.

^1^ Receptor classes are printed in bold, families in italics. ^2^ Abbreviations: act. B-L: activated B cell lymphoma cells; mon. ph.: mononuclear phagocytes; M1 Mθ, M2 Mθ: macrophages of different polarization. ^3^ Blood levels are expressed as multiples of the reference, followed by indications of significance levels of the changes and data source. ****: *p* < 0.0001; ***: *p* < 0.001; **: *p* < 0.01; *: *p* < 0.05; n.s.: not significant; n.d.: no data available.

## 4. T Cell Cytokines

In CLL patients, the dialogue with lymphoma cells creates an increased and skewed T cell compartment resulting in a state of immune suppression [28]. Owing to this shift in T cell subtype composition, culture supernatants of isolated phytohemagglutinin-activated T cells from CLL patients, but not from healthy donors, contain detectable levels of IL-4 [22]. According to enzyme-linked immunoassays, the supernatants of anti-CD3-activated T cells from healthy donors cultured in vitro contain the cytokines IL-6, IL-10 and tumor necrosis factor α (TNFα), but not IL-4 [21]. The PI3Kδ inhibitor idelalisib reduces the production of the detected T cell cytokines in a concentration-dependent manner [21]. Other cell types are also capable of producing these cytokines. For instance, adherence-primed mononuclear phagocytes after stimulation with lipopolysaccharide secrete TNFα [25] and CLL cells after stimulation with CD40 ligand the immunosuppressive cytokine IL-10 [23]. These two cytokines also serve to distinguish the inflammatory or immunosuppressive phenotypes of M1 or M2 polarized macrophages [24]. Treatment with ibrutinib strongly reduces the blood levels of these T cell cytokines in CLL patients, which are higher than in healthy controls apart from IL-10 (Table 1).

## 5. Myeloid Bystander Cells

A decisive role of the myeloid compartment in the pathogenesis of CLL is demonstrated in the first place by mouse models [15,29,30], but also supported by in vitro studies. Colony stimulating factor 1 (CSF-1), also known as macrophage CSF, plays decisive roles in the development of macrophages including their survival, proliferation, chemotaxis and polarization to an immunosuppressive phenotype [31,32]. Predominant secretion of IL-10 compared to TNFα reflects the immunosuppressive M2 polarization of tumor-associated macrophages [24]. So-called nurse-like cells were likened to thymic nurse cells owing to morphological similarity in their first description [14]. These cells that efficiently support the survival of CLL cells are derived from blood monocytes [33] and called lymphoma-associated macrophages in B cell malignancies other than CLL [34].

CSF-1 can be produced and secreted by adhering phagocytes but also by mantle cell lymphoma (MCL) cells [25,26]. Ibrutinib abrogates CSF-1 and IL-10 production in MCL cells and consequently inhibits macrophage polarization. The combination of BTK and CSFR inhibition leads to mutually enhanced effects on co-cultures of MCL cells and lymphoma-associated macrophages [26]. CSF-1 shows comparatively strongly elevated blood levels in CLL patients compared to healthy donors [27].

## 6. Conclusions

Enhancing a recent review article published in this journal [1], the present discussion of paracrine interactions in the B cell lymphoma microenvironment emanates from evidence gained with in vitro cultures of different cell types. The chemokines CCL3 and CXCL12 represent contrasting examples of induced and constitutive production by tumor and bystander cells. The cytokines IL-4, IL-6, IL-10 and TNFα are produced by T cells, but often also by activated lymphoma cells or macrophages. As an additional important cytokine in this context, we suggest the RTK ligand CSF-1 that is essential for the development of lymphoma-associated macrophages. Production and effects of many of these cytokines are subject to disturbance by inhibitors of BAK in B cell lymphoma cells and in different types of bystander cells. The roles assigned to these example cytokines in the micro-environmental dialogue partially explain the compiled alterations in the blood levels of the corresponding cytokines owing to CLL and their normalization upon treatment with the BTK inhibitor ibrutinib. Finally, our commentary may support informed judgement on the article by Allegra et al. [1].
